# The Impact of Social Determinants of Health on Supportive and Palliative Care in Pancreatic Cancer Management: A Narrative Review

**DOI:** 10.3390/cancers17193254

**Published:** 2025-10-08

**Authors:** Sterre van Herwijnen, Vishnu Jayaprakash, Camila Hidalgo Salinas, Joseph R. Habib, Daniel Brock Hewitt, Greg D. Sacks, Christopher L. Wolfgang, Katherine A. Morgan, Brian J. Kaplan, Michael D. Kluger, Alok Aggarwal, Ammar A. Javed

**Affiliations:** Division of Hepatobiliary and Pancreatic Surgery, Department of Surgery, NYU Langone School of Medicine, New York, NY 10016, USA; sterre.vanherwijnen@nyulangone.org (S.v.H.); vishnu.jayaprakash@nyulangone.org (V.J.); camila.hidalgosalinas@nyulangone.org (C.H.S.); joseph.habib@nyulangone.org (J.R.H.); brock.hewitt@nyulangone.org (D.B.H.); greg.sacks@nyulangone.org (G.D.S.); christopher.wolfgang@nyulangone.org (C.L.W.); katherine.morgan@nyulangone.org (K.A.M.); brian.kaplan@nyulangone.org (B.J.K.); michael.kluger@nyulangone.org (M.D.K.); alok.aggarwal@nyulangone.org (A.A.)

**Keywords:** pancreatic neoplasms, pancreatic cancer, social determinants, supportive care, palliative care, end-of-life care

## Abstract

**Simple Summary:**

Pancreatic cancer is one of the deadliest solid organ malignancies, with a 5-year survival rate of 13% across all stages of disease. Supportive and palliative care are critical aspects of patient management and can be impacted by social determinants of health (SDOH), such as income, education, and access to healthcare. This review demonstrates that racial disparities exist in receipt of pain medication and nutritional care, educational disparities in terms of accessing psychological support, and that end-of-life planning is less common among non-White and less-educated patients. These gaps also extend to rehabilitation services and telehealth. High-volume treatment centers show better outcomes for all patients, but minorities and low-income patients are less likely to receive care there. Addressing these disparities requires coordinated efforts at clinical, organizational, and policy levels to ensure equitable access to care for all pancreatic cancer patients.

**Abstract:**

Background: Pancreatic cancer is a challenging malignancy with an aggressive biology and limited treatment options, contributing to low survival rates. Supportive and palliative care play a key role in improving the quality of life and psychological distress for patients and their families. However, appropriate delivery and effectiveness of these interventions may be influenced by social determinants of health (SDOH). These factors create significant barriers for patients, influencing their access to care and ability to make informed decisions. This review explores the role of SDOH in supportive and palliative care of pancreatic cancer patients and identifies areas for improvement to enhance this type of care for vulnerable populations. Methods: A thorough narrative review was carried out to evaluate the influence of social determinants of health on supportive and palliative care in the management of pancreatic cancer, focusing on symptom management, psychosocial support, nutritional support, advance care planning, rehabilitation, functional support, and care coordination. Results: This review demonstrates that disparities exist. Black and Asian patients receive less pain medications; those with lower level of education struggle to access psychological support; Hispanic and Black patients often do not receive needed nutritional care; and end-of-life planning is less common among non-White and less-educated patients. Conclusions: SDOH significantly affects the experience and delivery of supportive and palliative care in pancreatic cancer patients, exacerbating inequities across multiple domains of care. Addressing these disparities requires coordinated efforts at clinical, organizational, and policy levels to ensure equitable access to care for all patients in their final phase of life. Integrating attention to SODH into care delivery models can improve outcomes and enhance quality of life for these patients.

## 1. Introduction

Pancreatic cancer remains one of the most challenging malignancies to manage, characterized by a poor prognosis and a complex treatment landscape involving coordination of multidisciplinary care. Despite advancements in medical therapies, survival rates for pancreatic cancer remain dismal, with a five-year survival rate of 13% in the United States [1]. Given the nature of the disease, supportive and palliative care have become essential components of treatment, with a focus on symptom management, providing psychosocial support, nutritional support, advance care planning (ACP), rehabilitation, functional support and care coordination. These interventions aim to improve quality of life (QoL) and psychological well-being of pancreatic cancer patients and their families [2].

Social determinants of health (SDOH), such as socioeconomic status (SES), access to healthcare, education, safe housing, and social support networks, play a crucial role in shaping the health outcomes of patients with pancreatic cancer [3,4]. For instance, patients from lower socioeconomic backgrounds may face barriers such as limited access to healthcare resources, financial hardship and social isolation, all of which can hinder the patient from receiving adequate supportive and palliative care [5]. Pancreatic cancer patients might also face challenges in physician-patient communication due to poor health literacy and gaps in understanding of the illness. This can restrict patients from making well-informed decisions that align with their goals and preferences for palliative care [6].

This narrative review seeks to summarize and synthesize the existing literature on the impact of social determinants on supportive and palliative care for pancreatic cancer patients. Additionally, it aims to identify specific target areas for improvement that could enhance the quality of life of vulnerable patient populations. Understanding these intersections is critical for developing more patient-centered care approaches that address all the components of the needs faced by pancreatic cancer patients.

## 2. Methods

A narrative literature search was conducted, ending in April 2025, to explore the role of social determinants of health (SDOH) in supportive and palliative care for patients with pancreatic cancer. Searches were performed in PubMed, Embase, and Google Scholar using combinations of terms related to “*pancreatic cancer*”, “*palliative care*”, “*supportive care*”, “*social determinants of health*”, and “*disparities*.” Only peer-reviewed, English-language publications were included, when full text was available.

An inverted funnel approach was used. For each SDOH domain, studies specific to supportive or palliative care in pancreatic cancer were first identified. When such evidence was not available, the scope was expanded to studies about supportive and palliative care in other gastrointestinal malignancies. This approach allowed for a comprehensive but targeted synthesis, prioritizing condition-specific evidence while contextualizing findings within the wider oncology literature. Additional references were identified through manual review of bibliographies of key articles.

## 3. Supportive and Palliative Care

### 3.1. Symptom Management

One of the most important aspects of supportive and palliative care in pancreatic cancer involves the management of symptoms. Although developments in imaging have contributed to early detection of disease, allowing for diagnosis of asymptomatic patients, most patients present at later stages of pancreatic cancer with high tumor burden and debilitating symptoms. Effective supportive care not only alleviates symptoms but also reduces hospitalization and enhances QoL.

Approximately three-quarters of patients present with pain at the time of diagnosis, often influenced by the tumor’s location and the presence of local invasion or metastasis [7]. Patients with pancreatic cancer report higher rates of anxiety and depression compared to those with other cancer types. Between 15% and 40% of patients experience depressive symptoms, which can amplify pain and negatively impact QoL [8,9,10,11]. Biliary decompression is often necessary in advanced pancreatic cancer, with many patients experiencing endoscopic retrograde cholangiopancreatography (ERCP) due to cholangitis, delayed surgery, or relief of jaundice. It plays a pivotal role due to its association with fewer complications, shorter hospital stays, and improved QoL [12]. Inadequate management of cancer-related pain and other symptoms is a frequent contributor to treatment interruptions and has been linked to poorer clinical outcomes, highlighting the critical importance of supportive care in pancreatic cancer [13,14].

Supportive care plays a major role in managing symptoms and enhancing QoL in pancreatic cancer, yet its utilization remains uneven across socioeconomic and racial groups. An analysis of 74,309 patients from the Surveillance, Epidemiology and End Results (SEER)-Medicare linked database showed significant racial disparities in the use of supportive care medications. After adjustment for confounding factors, Black and Asian patients had lower utilization of opioids compared with Non-Hispanic Whites [Black adjusted odds ratio (aOR) 0.84, 95% CI 0.79, 0.88 and Asian aOR 0.84, 95% CI 0.79, 0.90] [15]. Conversely, the use of non-opioid analgesics was higher among Asian (aOR 1.37, 95% CI 1.26, 1.49) and Hispanic patients (aOR 1.16, 95% CI 1.01, 1.14) when compared to Non-Hispanic Whites, whereas no significant difference was observed between Black patients (aOR 0.97, 95% CI 0.90, 1.04) and Non-Hispanic Whites. Disparities extended to mental health medications. Relative to Non-Hispanic Whites, antidepressant utilization was markedly lower among Black (aOR 0.56, 95% CI 0.53, 0.59), Hispanic (aOR 0.77 95% CI 0.73, 0.82), and Asian patients (aOR 0.47, 95% CI 0.44, 0.51). Similar trends were observed for anxiolytics (Black: aOR 0.78, 95% CI 0.74, 0.82; Hispanic: aOR 0.66, 95% CI 0.62, 0.71; Asian: aOR 0.52, 95% CI 0.48, 0.57), and antipsychotics (Hispanic: aOR 0.90, 95% CI 0.82, 0.99; Asian: aOR 0.84, 95% CI 0.74, 0.95). These findings suggest that Black, Asian, and Hispanic patients received less pharmacological supportive care than Non-Hispanic Whites. This may stem from factors such as limited access to care and pain specialists, miscommunication about pain severity, differing patient beliefs about analgesics, and provider biases or knowledge gaps regarding patient pain [16]. There is evidence indicating that pharmacies located in predominantly minority neighborhoods are less likely to maintain an adequate supply of opioids, potentially contributing to disparities in pain management [17,18]. In one national survey, more than half of responding pharmacies reported inadequate supplies of opioids to treat patients with severe pain [18]. The problem is particularly pronounced in predominantly non-White neighborhoods, where only one in four pharmacies carried sufficient opioid medications, compared to nearly three-quarters of those in predominantly White neighborhoods (*p* < 0.001). A separate analysis from Michigan reinforced these findings; pharmacies located in predominantly White zip codes were substantially more likely to stock adequate opioid supplies than those in minority zip codes, with odds ratios (OR) ranging from 13.36 (95% CI 1.09, 164.17) in higher income areas to 54.42 (95% CI 6.27, 472.02) in lower income areas [17]. Collectively, these findings highlight systemic inequities in accessing essential pain medications.

Disparities are also evident in the use of biliary interventions among patients with pancreatic cancer. Although ERCP remained the most common approach to biliary decompression, utilization patterns vary across patient subgroups. Black patients were significantly less likely than White patients to undergo ERCP (aOR 0.84, 95% CI 0.72, 0.97) and more likely to undergo percutaneous transhepatic biliary drainage (PTBD) (aOR 1.38, 95% CI 1.14, 1.66) [19]. Beyond race, other demographic variables have also been shown to influence ERCP utilization. Older individuals (aOR 0.88, 95% CI 0.83, 0.94), unmarried individuals (aOR 0.92, 95% CI 0.86, 0.98), and individuals residing in rural areas (aOR 0.89, 95% CI 0.82, 0.98) were also associated with lower rates of ERCP [20].

### 3.2. Psychological and Social Support

Besides the significance of symptom management in supportive and palliative care in pancreatic cancer patients, psychological and social support are essential. The low curability and survival rate of pancreatic cancer have a profound impact on patients’ QoL, often leading to significant deterioration. This is particularly evident in changes in mental health, cognitive functions, and the ability to cope with the disease [21]. Consequently, psychological and social support are crucial in this context. Micheal et al. present evidence highlighting the importance of timely referral to palliative care for pancreatic cancer patients, emphasizing that a lack of timely access often results in inappropriate end-of-life treatment, which negatively impacts psychological well-being. Patients receiving late palliative care (<90 days before death) were associated with 12.5% more acute hospital admissions and 18.1% more ED presentations [22]. Furthermore, findings from Chung et al. demonstrate that early palliative care improves the psychological resilience of patients and enhances their overall QoL. The intervention group showed trends toward improvement in the physical, social, and emotional aspects of the Functional Assessment of Cancer Therapy-General (FACT-G) subscales, as well as reduced psychological distress from baseline to 12 weeks, compared to the pancreatic cancer care patients receiving usual care [2].

Osagiede et al. state that SDOH, such as gender, age, insurance status, and comorbidity, play a role in facilitating or hindering access to needed psychological support services in pancreatic cancer patients receiving supportive and palliative care. Palliative care recipients were more likely to be older, Medicaid-insured, and nonobese, while males, Medicare-insured individuals, those with a lower Charlson comorbidity score, and patients treated in urban nonteaching hospitals were less likely to receive it [23].

The various SDOH can differently impact various components of supportive or palliative care, from understanding the diagnosis, to seeing the importance of psychological support, to eventually receiving the needed mental health services. Lower educational attainment can hinder a patient’s comprehension of their diagnosis and the importance of psychological support, leading to underutilization of mental health services, which can ultimately result in poorer mental health outcomes [24]. Additionally, patients with lower SES often face financial barriers that limit access to psychological support services, such as counseling and mental health medications [25]. Not only this, but financial stress can exacerbate psychological distress, impacting overall well-being. Moreover, living in underserved or rural areas can restrict access to mental health professionals and supportive care services [26]. Poor living conditions and high-crime neighborhoods can also increase stress and anxiety levels.

Furthermore, research shows that social support is a significant mediator in the psychological distress experienced by individuals with serious health issues in general. For instance, Bøen et al. compared psychological distress and social support within adults. They suggest that low socioeconomic conditions correlate with reduced social support, which increases psychological distress among older adults. This observation is pertinent for pancreatic cancer patients, who often face both physical and emotional burdens [27]. Studies confirm that robust social support networks can buffer psychological stress, thereby enhancing patient resilience during challenging medical treatments [22,28]. In line with these findings, Yang et al. convey that perceived social support may play a vital role in encouraging patients to seek professional psychological help, indicating that stigma surrounding mental health can adversely impact one’s willingness to access necessary support services [29].

### 3.3. Nutritional Support

Weight loss is seen in up to 80% of patients with pancreatic cancer, often driven by malnutrition and cancer related cachexia [30]. This is closely associated with worse clinical outcomes, including decreased survival, disease progression, unplanned hospital admissions, limited tolerance to treatment, and decreased QoL [31,32]. While the exact mechanisms of cancer-associated cachexia remain unclear, growing evidence links it to metabolic disturbances, particularly elevated energy expenditure and enhanced proteolysis driven by cellular pathways such as ubiquitin-proteasome activity and autophagy [33]. Research has shown that nutritional support, ranging from dietary supplements to parenteral or enteral nutrition, is a key component of a comprehensive early palliative care approach and can significantly contribute to improving QoL [32]. Early nutritional interventions, including dietary modifications and supplementation, can help mitigate these adverse outcomes. Exocrine pancreatic insufficiency (EPI) is commonly seen in pancreatic cancer resulting in malabsorption and malnutrition. EPI adversely affects QoL and has been associated with poor survival [34,35]. Pancreatic enzyme replacement therapy (PERT) is the cornerstone of managing EPI, helping to restore nutrition and reduce gastrointestinal symptoms.

A recent study involving patients undergoing pancreatectomy noted that Hispanic patients had a higher proportion of cachexia compared to other racial groups [36]. In a study involving patients with gastrointestinal cancer, after controlling for confounders, Black and Hispanic racial groups were at higher risk of presenting with cachexia (Black aOR 2.447, 95% CI 1.62, 3.697, and Hispanic aOR 3.039, 95% CI 1.943, 4.754) compared to Non-Hispanic White patients [37]. The absence of private insurance was also associated with higher risk of cachexia as compared to patients with private insurance. Recent evidence suggests disparities in PERT prescribing that disproportionately affect African American (OR 0.7281, 95% CI 0.6628, 0.7998) and older patients (OR 0.8064, 95% CI 0.7604, 0.8551) with pancreatic cancer [38].

In another study by Latenstein et al., it was noted that although two-thirds of the patients had cachexia, only half of the patients received dietitian consultation [31]. This highlights the need for improved referral rates and access to dietitian services. EPI remains underdiagnosed and undertreated in pancreatic cancer [39].

### 3.4. Advance Care Planning (ACP)

To provide optimal supportive and palliative care, the implementation of advance care planning (ACP) is essential. ACP is a process through which patients, together with their healthcare professionals, family members, and important others, make decisions on their future medical treatment and end of life care [40]. Oncologists and palliative care specialists share the responsibility of openly discussing prognosis and suggesting end-of-life care options at appropriate stages of a cancer patient’s disease progression. During these patient-centered conversations, physicians must be sensitive to the individual’s unique cultural, spiritual, and moral values that influence decision-making, while acknowledging that these decisions are dynamic and may change over time. The primary goal of ACP is to help patients reflect on their end-of-life goals, enabling them to make informed healthcare decisions that align with and fulfill these preferences. For patients with incurable cancers, such as unresectable or metastatic pancreatic cancer, ACP is a crucial aspect of oncologic care and should be regularly addressed alongside treatment recommendations [6]. For patients who receive end-of-life care in general, research has shown that the presence of ACP discussions in these patients is associated with lower rates of ventilation, resuscitation, intensive care unit admission, earlier hospice enrollment, and reduced cost of care at the end of life [41,42].

The ACP process offers an opportunity for healthcare providers and patients to discuss relevant SDOH, allowing these factors to be documented as part of the ACP. This may support the development of more sustainable and personalized ACP [43]. Research by Spelten et al. highlights the impact of SDOH on ACP in all cancer patients. In specific, they focus on eleven studies, which cover two overarching themes: person-related factors (e.g., socio-demographic characteristics), and comprehension and awareness. White, well-educated patients with a support network were found to be more likely to engage in ACP. However, many cancer patients have limited comprehension and awareness of ACP, which may affect their involvement in decision-making about end-of-life care [44].

LoPresti et al. present, in a systematic review including 25 articles, that hospice care (specific type of palliative care) for cancer patients in general was mostly used among Whites, and least by African and Asian Americans. Despite this, the need for hospice care among the last group was greater and yet more frequently they had inadequate knowledge about their options for end-of-life care. Additionally, end-of-life care provided to African Americans was often inconsistent with patients’ preferences, an important component discussed during ACP conversations [45]. Other research shows that determinants such as age, gender, geographic location, preference for aggressive care, and knowledge of hospice impacts the use of hospice care in African American patients with cancer [46].

Agarwal and Epstein’s review discusses the use of palliative care and advance care planning for patients with pancreatic and other cancers. They emphasize that obstacles in physician–patient communication, such as poor health literacy, limited time in outpatient settings, inaccurate prognostic expectations, and gaps in understanding the illness, can hinder patients from making well-informed decisions that align with their goals and preferences for future care [6]. SODH such as education, language and literacy skills, and transportation can further exacerbate these challenges. Practical strategies to address these barriers include the use of professional interpreters and culturally appropriate ACP tools, which can support equitable patient engagement in decision-making.

### 3.5. Rehabilitation and Functional Support

The rapid physical decline commonly associated with pancreatic cancer necessitates timely and appropriate rehabilitation interventions to maintain QoL, independence, and dignity throughout the palliative phase whenever possible. Patients with pancreatic cancer frequently experience profound functional limitations due to disease progression, treatment side effects, and nutritional compromise. Therefore, palliative rehabilitation focuses on patient comfort [47]. Rehabilitative support enables patients to sustain regular physical activity throughout periods of disease stability or ongoing treatment, while palliative rehabilitation approaches aid preservation of optimal quality of life as patients transition to end-of-life care. Physical activity and exercise interventions remain valuable contributors to patient well-being regardless of disease progression or treatment phase, serving as consistent components of comprehensive supportive care [47]. Ibrahim et al. emphasize the impact of rehabilitation integrated in a palliative program on the QoL of patients with terminal cancer. Their program for rehabilitation palliative care enhanced the overall QoL among patients and their caregivers, and it reduced anxiety and depression levels [48].

Existing research on the impact of SDOH in rehabilitation and functional support in these patients is sparse. A scoping review from Marc Sempedro Pilegaard et al., including 11 studies, aimed to map existing research of rehabilitation and palliative care for socioeconomically disadvantaged patients. The review shows that financial constraints are perceived as barriers to utilizing palliative care for cancer patients; however, none of their included studies addressed rehabilitation, and the results of the studies were to some extent contradictory [49]. Elk et al. present that, in diverse, highly vulnerable groups, such as immigrants, homeless populations, and disabled individuals, the lack of access to palliative care is evident. Additionally, they mention the lack of research to create palliative care programs which meet the needs of every separate vulnerable group [50].

### 3.6. Care Coordination and Telehealth

The management of pancreatic cancer requires complex, multidisciplinary collaboration spanning gastroenterology, surgical oncology, medical oncology, radiation oncology, nutrition, psychosocial support services, and palliative care [51]. Care coordination is a fundamental element of supportive and palliative care in these patients. Effective care coordination not only streamlines navigation through complex treatment options but also enhances continuity of care, which has a profound impact on patient outcomes. Given the aggressive nature of pancreatic cancer, which is marked by a rapid decline in patient health and QoL, ensuring that patients receive timely and well-coordinated care is essential for addressing their multifaceted needs [52,53]. Addressing SDOH is critical in optimizing care coordination. Factors such as SES, access to transportation, and support networks significantly influence a patient’s ability to engage with care services and adhere to treatment protocols [54]. By accommodating these determinants within care models, healthcare providers can enhance patient participation, enable timely interventions, and ultimately contribute to better health outcomes [23,55].

Wolfson et al. demonstrated that patients receiving care at high-volume centers with established multidisciplinary teams experienced improved survival outcomes and QoL compared to those treated at centers without formalized care coordination. They also state that barriers to care at the National Cancer Institute-designated comprehensive cancer centers included race/ethnicity, insurance status, SES, and distance to a given center [56].

Digital divides further exacerbate care coordination challenges. While telehealth has emerged as a promising tool for improving care coordination, Nouri et al. revealed significant disparities in digital literacy and technology access among patients, noting that older patients, those with lower incomes, and racial/ethnic minorities faced greater barriers to telehealth utilization [57]. More recent evidence supports that these disparities in telehealth access persist. For example, a study by Everson et al. found that education level, broadband access, and geographic location remained significantly associated with whether telehealth was offered among adults [58]. Another recent analysis by Marcondes et al. showed racial/ethnic minorities were less likely to receive telemedicine, after adjusting for clinical and geographic variables [59].

## 4. Discussion

This review explores the impact of SDOH on supportive and palliative care for patients with pancreatic cancer, revealing significant disparities across multiple domains of care. Supportive and palliative care are critical aspects of managing pancreatic cancer, yet they are often overlooked. While early integration of supportive and palliative care is becoming more common, gaps remain in ensuring equitable access for all patients [60,61,62]. These interventions not only enhance QoL but are also associated with improved survival outcomes and greater adherence to treatment [63]. They encompass a wide range of multidisciplinary interventions, including pain and symptom management, treatment of depression and anxiety, psychological and nutritional support, advance care planning, rehabilitation, and coordination of care. Importantly, a thoughtful and patient-centered approach is essential to balance the risks of undertreatment with the harms of overly aggressive interventions at the end of life.

**Figure 1:**
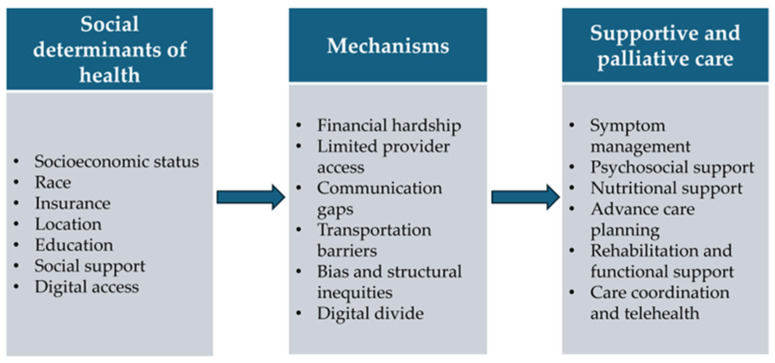
SDOH driving disparities in supportive and palliative care in pancreatic cancer.

A recent study on supportive care in patients with locally advanced pancreatic cancer revealed that, despite most patients receiving care at multidisciplinary centers and residing in metropolitan areas near their primary treatment facility, fewer than 10% received any supportive care [63]. SDOH significantly influences the quality, accessibility, and utilization of supportive and palliative care in pancreatic cancer. Across symptom management, disparities persist in the use of opioids and procedures such as ERCP, with Black and Asian patients experiencing lower rates of opioid prescriptions and ERCP utilization. These inequities are often driven by limited access to specialized care and structural barriers such as limited pharmacy availability in minority neighborhoods. Similarly, psychological and social support services are underutilized among racial and ethnic minorities, patients with lower educational attainment, and those with public insurance or who reside in rural areas. These populations frequently face barriers including reduced health literacy, stigma, and limited availability of mental health professionals, all of which contribute to unmet psychosocial needs. Nutritional support, a critical component of palliative care, is also marked by disparities; Hispanic and Black patients are disproportionately affected by cachexia and are less likely to receive timely dietitian consultations. ACP is less commonly pursued by individuals with lower SES, limited health literacy, or non-White racial backgrounds, despite evidence linking the presence of ACP to improved end-of-life outcomes. These disparities are exacerbated by communication barriers and insufficient culturally sensitive practices. Rehabilitation and functional support services remain largely inaccessible to marginalized populations, including immigrants and individuals with disabilities, due to financial and systemic barriers. Finally, although care coordination and telehealth have the potential to improve access, their effectiveness is curtailed by geographic, technological, and socioeconomic gaps that disproportionately impact racial minorities, older adults, and low-income individuals. As illustrated in Figure 1 and detailed in Table 1, these disparities extend across nearly all supportive care domains, from pain management and psychosocial services to nutrition and care coordination. Addressing these multifaceted inequities is essential for delivering equitable and comprehensive supportive care in pancreatic cancer. Additionally, further research is urgently needed to better understand the nuanced impact of SDOH across diverse populations and to develop targeted interventions particularly in understudied domains such as rehabilitation, nutritional support, and culturally tailored advance care planning. Moreover, most studies investigate disparities in supportive and palliative care with a focus on individual-level social factors, with less attention to community and structural determinants. Future research should adopt multilevel frameworks, such as neighborhood characteristics, healthcare system structures, and broader social policies.

Multiple studies have demonstrated that racial minority groups experience worse outcomes in pancreatic cancer. However, treatment at high-volume centers has been associated with improved outcomes among the marginalized populations [64]. Similarly, patients from lower SES backgrounds also show better survival when treated at high-volume centers [65]. These disparities may, in part, be explained by the poorer outcomes typically observed at low-volume centers, where racial and socioeconomic minority groups are disproportionately more likely to receive care [66,67].

Healthcare access remains a critical determinant in addressing disparities in outcomes associated with SES. Evidence indicates that reducing financial barriers and expanding health insurance coverage can significantly lower gastrointestinal cancer related mortality among racial minority populations [68]. Moreover, expansion of insurance coverage has been associated with reduced travel distances to care, which is particularly important for patients in rural or underserved areas [69]. Utilization of palliative therapy among patients with metastatic pancreatic cancer varies significantly by SES. Notably, Medicaid expansion has been associated with increased access to and use of palliative care services in this population [70]. While the adoption of telehealth accelerated during the COVID-19 pandemic, it also underscored persistent disparities in digital access and utilization, disproportionately affecting socioeconomically disadvantaged groups. Although the ubiquity of mobile phones has helped improve access, barriers such as digital literacy and reliable internet access continue to hinder effective telehealth use for some patient cohorts [71]. It is imperative for healthcare providers to acknowledge these limitations and adapt care models accordingly. To further mitigate disparities, efforts should focus on regionalizing care and establishing robust referral networks that facilitate timely access to high-quality supportive services, particularly in geographically and socioeconomically disadvantaged communities.

Community health workers (CHWs) have proven effective in bridging care gaps among underserved demographic groups. Patel et al. demonstrated that a comprehensive employer health-fund-based intervention utilizing CHWs significantly improved QoL, reduced frequent ED visits, and lowered overall healthcare costs for low income and racial minority patients [72]. Notably, the Centers for Medicare & Medicaid Services (CMS) has introduced a new billing code for health system coordination which will promote CHWs integration in delivering care [73]. In parallel, national organizations such as the Pancreatic Cancer Action Network (PanCAN), National Pancreas Foundation, Nikki Mitchell Foundation, and Hope Suite Hope play an important role in supporting access to supportive and palliative care. These organizations offer resources such as financial assistance, patient navigation, education, and psychosocial support. These services help patients, particularly those in underserved and rural communities, navigate complex care pathways. Support groups led by these organizations also emphasize continued symptom management, nutritional support, and mental health care, further extending the impact of supportive care. By collaborating with healthcare systems and engaging in policy advocacy, these organizations complement the efforts of CHWs in addressing disparities and improving care equity for patients with pancreatic cancer. Additionally, ensuring the participation of racial and socioeconomically diverse groups in clinical trials is essential to improve the generalizability of findings and addressing disparities in treatment outcomes. Evidence indicates that pancreatic cancer trials are affected by the underrepresentation of various racial minorities [74]. Addressing this gap requires strategic efforts, including the establishment of accessible trial sites, recruiting culturally competent teams, fostering unbiased environments, and supporting sustained advocacy efforts.

## 5. Conclusions

SDOH profoundly impact the experience and efficiency of supportive and palliative care for patients with pancreatic cancer, reinforcing inequities across multiple domains of care. Addressing these disparities requires coordinated efforts from different levels (clinical, organizational, and policy) to ensure that all patients, regardless of their background, receive the needed supportive or palliative care throughout their care. By integrating attention to this matter into supportive care delivery models, healthcare systems can move toward more equitable outcomes and improved QoL for patients facing this challenging diagnosis.

Future studies should focus on multilevel approaches that address structural and community-level determinants, while also targeting underexplored areas such as rehabilitation, nutrition, and culturally tailored advance care planning. Efforts to expand diverse trial participation and evaluate interventions like telehealth and community health workers are crucial to achieving more equitable supportive and palliative care. In parallel, strengthening health system infrastructure by regionalizing care and building robust referral networks can help ensure that patients in geographically and socioeconomically disadvantaged communities have timely access to high-quality supportive services.

## Figures and Tables

**Table 1:** Summary of disparities in supportive and palliative care for pancreatic cancer.

Domain of Care	SDOH	Affected Group	Observed Disparity	Study
Symptom management	Race	Black, Asian, and Hispanic patients	Opioids: Lower utilization among Black: aOR 0.84 (95% CI 0.79–0.88) and Asian: aOR 0.84 (95% CI 0.79–0.90) patients compared with Non-Hispanic Whites; Antidepressants: Lower utilization among Black aOR 0.56 (95% CI 0.53–0.59), Hispanic aOR 0.77 (95% CI 0.73–0.82) and Asian patients aOR 0.47 (95% CI 0.44–0.51) compared with Non-Hispanic Whites.	Allen et al., [15]
	Race, location	Non-White neighborhoods	Opioid availability: Stocked in only 25% of pharmacies in predominantly non-White neighborhoods vs. 72% in White neighborhoods.	Morrison et al., [18]
	Race, location	Minority neighborhoods	Opioid availability: Adequate in 54% of pharmacies in minority neighborhoods vs. 86.9% in White neighborhoods.	Green et al., [17]
	Race	Black patients	ERCP utilization: Lower among Black patients (aOR 0.84 (95% CI 0.72–0.97)) compared to white patients.	Tavakkoli et al., [19]
	Age, marital status, location	Older patients, unmarried, and rural area	ERCP utilization: Lower among older individuals (aOR 0.88 (95% CI 0.83–0.94)), unmarried individuals, (aOR 0.92 (95% CI 0.86–0.98)), and rural residents (aOR 0.89 (95% CI 0.82–0.98))	Rustgi et al., [20]
Psychological and social support	Access to palliative and supportive care	Patients receiving late palliative care	Inappropriate end-of-life care impacted psychological well-being. Comparing early to late palliative care referral, the second group had more ED presentations (18.1% (95% CI 6.8–29.4%)) and more acute hospital admissions (12.5% (95% CI 1.7–24.8%)).	Michael et al., [22]
		Patients receiving late palliative care	Early palliative care may improve QoL and psychological distress: 26 pancreatic cancer patients received advanced practice nurse driven palliative care intervention versus 16 patients receiving normal care. A positive impact on treatment, potentially leading to longer survival, was observed in the intervention arm.	Chung et al., [2]
	Gender, age, insurance status, comorbidity	Younger, male, Medicare insured, lower Charlson comorbidity score	Retrospectively (22,053 pancreatic cancer patients), less access to psychological support services.	Osagiede et al., [23]
	SES	Low SES patients	Association between lack of social support and psychological distress. Even and indicated direct effect.	Bøen et al., [27]
Nutritional support	Race	Black and Hispanic patients	Cachexia risk: Higher in Black (aOR 2.447 (95% CI 1.62–3.697)) and Hispanic patients (aOR 3.039 (95% CI 1.943–4.754)) vs. Non-Hispanic White patients.	Olaechea et al., [37]
	Race	African American patients, older patients	PERT utilization: Less likely in African Americans (OR 0.7281 (95% CI 0.6628–0.7998)) and older patients (OR 0.8064 (95% CI 0.7604–0.8551)).	Chittajallu et al., [38]
Advance Care Planning (ACP)	Race, education, support network	Non-White, less-educated, no support network	White, well-educated patients with a support network were more likely to be involved in ACP. However, within cancer patients, there is limited comprehension regarding ACP.	Spelten et al., [44]
	Race	African and Asian Americans	Higher need for hospice care, yet more frequently inadequate knowledge. Inconsistency with the patients’ preferences or inadequate documentation of their ACP involving religious or spiritual beliefs.	LoPresti et al., [45]
	Education, language, literacy skills	Less-educated, language barrier	Poor health literacy, limited time in outpatient settings, inaccurate prognostic expectations, and gaps in understanding can be an obstacle in making well-informed decisions, considering all the preferences and wishes for the patients’ care goals.	Agarwal and Epstein [6]
Rehabilitation and functional support	SES	Patients with financial constrains	A barrier to utilizing palliative care.	Marc Sempedro Pilegaard et al., [49]
	Immigration status, SES, disability	Immigrants, homeless, disabled	Lack of access to palliative care and lack of research to meet the needs of these vulnerable groups.	Elk et al., [50]
Care coordination and telehealth	Access to care	Patients treated at centers without formalized care coordination	Centers with established multidisciplinary teams experience improved survival outcomes and QoL. Barriers to receiving care at these centers are race/ethnicity, insurance status, SES, and geography.	Wolfson et al., [56]
	Age, SES, race	Digital literacy and technology access	Barriers to telehealth utilization.	Nouri et al., [57]

## Data Availability

No new data were generated.

## Abbreviations

The following abbreviations are used in this manuscript:

SDOH	Social determinants of health
aOR	Adjusted odds ratio
PTBD	Percutaneous transhepatic biliary drainage
SES	Socioeconomic status
HPA	Hypothalamic–pituitary–adrenal
ERCP	Endoscopic retrograde cholangiopancreatography
ED	Emergency department
QoL	Quality of life
EPI	Exocrine pancreatic insufficiency
PERT	Pancreatic enzyme replacement therapy
ACP	Advance care planning
CHWs	Community Health Workers
PanCAN	Pancreatic Cancer Action Network
